# Reshaping Anesthesia with Artificial Intelligence: From Concept to Reality

**Published:** 2025-09-08

**Authors:** Aleena Dost, Raneem Alaraj, Rabeeya Mayet, Devendra K Agrawal

**Affiliations:** Department of Translational Research, College of Osteopathic Medicine of the Pacific, Western University of Health Sciences, Pomona, California, USA

**Keywords:** Anesthesia, Anesthesia information management system, Anesthesiology, Artificial intelligence, Deep learning model, Machine learning model, Pharmacogenomics

## Abstract

Artificial intelligence (AI) is transforming anesthesiology, showcasing applications that address patient monitoring, closed-loop anesthetic delivery, risk forecasting, customized management, and workflow betterment. This review highlights modern developments, analyzing the role of AI from early rule-based systems to machine learning and deep learning models, aided by the foundational role of anesthesia information Management Systems. AI processes depict strong performance of the clinical team and allowing anesthesiologists to intervene earlier in cases of intraoperative hypotension, acute kidney injury, tissue hypoxia, and giving them more time to focus on complex patient cases. Closed-loop systems guided by the physiologic and electroencephalogram feedback exemplify the ability of AI to maintain anesthetic stability while reducing clinician workload. Predictive models help with the American Society of Anesthesiologists’ classification in categorizing the patients, airway risk stratification, and customized treatment planning with improving preoperative evaluation. A move toward precision anesthetic administration is indicated by new developments in pharmacogenomics, perioperative pain characterization, and AI-assisted ultrasonography. Beyond clinical gains, AI guarantees improved operating room efficiency through organized scheduling, natural language processing documentation. Yet, widespread integration of AI in anesthesia still faces barriers regarding ethical concerns, clinical doubt, including replicability amongst healthcare systems, and a lack of in-depth data regarding the topic. Addressing these concerns demands data from multicenters, interdisciplinary education, and integration of explainable AI frameworks that are palatable to the clinical world. Overall, AI has the potential to behave as an adjunct, instead of replacing anesthesiologists by aiding in decision making, improving patient safety, and preparing for perioperative care.

## Introduction

Artificial intelligence (AI) is an offshoot of the technological world, faceted with the ability to perform tasks that typically require human-level capabilities. A recent bibliometric analysis examined about 658 AI-related publications in anesthesiology, showing a sharp increase in research post-2019, especially in areas like intraoperative hypotension prediction, AI-assisted ultrasound for regional anesthesia, and monitoring systems [1]. Indeed, the AI-based prediction algorithms and biofeedback tools enhance individualization of care by enhancing precision, reducing recovery time, and improving patient outcomes [2,3]. Its domains include machine learning (ML)—where algorithms progress from data without direct scripting, deep learning (DL)—which is a part of ML that uses neurologic connections to model the intricate patterns; and the neurologic connections themselves are interconnected, mimicking the formatting of the human brain to break down data [4]. We have used AI in the evaluating the patterns of electromagnetic field stimulation in the brain [5–7]. Researchers have expanded on these ideas, emphasizing how AI in healthcare compromises both virtual systems, such as machine learning indications, and physical systems, like robotics in surgery. Indeed, the AI implementation in healthcare is becoming critical in the diagnosis, surgical and non-surgical treatment, patient follow-up, and categorizing studies by their focus on patient education, surgical assistance, and outcome assessment [8,9]. It has also been discussed how current AI applications are troubled with unclear logic regarding neural networks [10].

The journey of healthcare providers in anesthesiology has progressed from simple tools (such as laryngoscopes) to machines (like infusion pumps and anesthesia workstations). It is now entering a phase of computerization, where intelligent systems, like modern ventilators, autonomously regulate patient care with minimal clinician input [11]. A dual closed-loop system that uses ECG feedback to control propofol and remifentanil infusions enhanced anesthetic stability and decreased manual intervention in actual patients, according to a clinical experiment conducted [12]. AI developments, such as machine learning, deep learning, and neural networks, have been delved into for real-time decision aid and custom pharmaceutical delivery in anesthetic care [13]. The need for reproducible, context-aware design is further supported by the fact that these models, which usually undergo a multistage development process, do not necessarily translate well across institutions universally [14].

In the contemporary medical world, AI is transforming how we diagnose, plan treatment, monitor patients, and make decisions across various specialties, including cardiology, radiology, orthopedic surgery, neurosurgery, and critical care [2–8]. It has been argued that these modern technologies showcase a shift in how clinicians will practice medicine, requiring reflection on the changing role of doctors as interpreters, collaborators, and ethical leaders in an AI-supported healthcare domain [15]. This concept has been reinforced by exploring how growing abilities of AI may redefine the position of an anesthesiologist—not just as a provider of anesthesia, but as a data analyzer, systems manager, and patient advocate in increasingly machine-focused environments [16]. From a non-American perspective, a national survey found that most Turkish anesthesiologists believe AI could minimize complications and improve training in regional anesthesia [17].

In the surgical realm and perioperative medicine, AI has the potential to drastically improve precision and minimize human errors [16]. In an overview of AI in surgery, the incorporation of machine learning in perioperative care is quickly adapting, with general implications for surgical precision, workflow efficiency, and risk control [18].

AI has already shown potential in areas such as intraoperative decision-making, foreshadowing of hemodynamic instability, and optimizing pharmacological resources, increasing its value across all phases of perioperative care [8,19]. Its expansive role now incorporates monitoring hemodynamic fluctuations, ventilator support cases, and predicting early risks in ICU and perioperative settings [20].

More specifically, AI could aid us in predicting analytics to guide procedures and enhance our clinical decision-making support systems. AI can reshape core areas of anesthetic care, from real-time intraoperative monitoring to early risk stratification and pharmacologic guidance [21]. Modern literature has also underscored how AI is improving precision and custom anesthetic care but is also beginning to change predictive trajectories by forecasting complications [22].

The field of anesthesiology is deeply dynamic, quick-paced, with a high demand for rapid critical decisions and constant physiologic measuring, making it a huge field for AI integration [23]. This specialty has a constantly evolving exchange between vital signs observation, pharmacological intervention, and intraoperative calibration. These core tasks of anesthesiology harmonize well with AI’s ability to recognize patterns and process data in real time [24]. AI has also shown potential in regard to airway management, by utilizing machine learning models to help in predicting complicated intubations based on facial structure, patient speech aspects, and patient form; these tools outshine typical bedside aides and could drastically change preoperative airway risk stratification [25]. More specifically, AI in anesthesiology is currently showing up in regional anesthesia, ventilator weaning probabilities, and monitoring during surgery.

Although AI has grown more popular in the layman world, numerous anesthesiologists are hesitant about the involvement of AI within the field as well as its true capacities. Surveys showcase a mix of hope and hesitation; the obstacles include a lack of awareness, concerns regarding ethics, and the logical integration challenges of AI into the workplace. Interestingly, while there have been AI tools that are great aids in the medical world, e.g., ultrasound image guidance for nerve blocks, their extensive implementation is quite limited by barriers in policy, education, and infrastructure. In a randomized trial, researchers utilized an AI-guided endoscopy system (ENDOANGEL) that gave real-time dosing requests during sedation; this reduced recovery time and risks without increasing propofol use, showing how real-time AI feedback can improve procedural sedation [26].

The lack of standardized evaluation consistency across institutions makes it more difficult to assess which AI models are safe and reliable, thus slowing their adoption in clinical workflows [27]. A few researchers argued that many modern AI models in acute care still lack true situational awareness and typically fail to deliver bedside usage due to challenges like incohesive data sources, unclear model outputs, and restricted clinician interaction in the development process [28]. Although imaging-based AI tools have shown positive promise in forecasting complex airways, it must be recognized that factors like cost, radiation exposure, and staff burden still hinder their routine use in clinical environments [29].

The goal of this article is to analyze contemporary applications of artificial intelligence in the field of anesthesiology and identify its potential to promote patient safety, clinical efficacy, and perioperative impacts. Scientists conducted an infometric study showcasing the increasing growth of AI-related studies in anesthesiology over the past ten years, depicting a surge in academic and clinical interest in the field [30]. The growing academic and clinical focus on the perioperative outcomes of AI warrants an organized approach to education and safe execution [31].

Building upon an expanding base of clinical research, surveys, and validation trials, this review will highlight the pros and cons of AI support as it is being integrated into the field of anesthesiology.

## Historical Perspective and Evolution of AI in Medicine

The implementation of artificial intelligence into the medical world has been consistently changing over the last decade, starting with early rule-based systems (MYCIN and INTERNIST), where the goal is to reproduce clinical reasoning to diagnose and treat patients. Early rule-based systems like MYCIN evolved into modern neural networks capable of predictive modelling in perioperative care; they describe a timeline from foundational AIMS (electronic systems used in the operating room), platforms, to current decision-support algorithms [32]. These basic core components of medicine lay the foundations for machine-supported healthcare decision-making. Modern progress in computing power, databases, and algorithmic design—particularly through continuous neural networks—has propelled AI’s information into medicine, with anesthesiology as an ideal candidate due to critical patient cases and continuous real-time feedback [33].

In the field of anesthesiology, a crucial step toward AI integration was the addition of the Anesthesia Information Management Systems (AIMS). These systems rose to fame in the late 20^th^ century to computerize intraoperative documentation, capturing vital signs, medication administration information, and anesthetic parameters. By standardizing perioperative data, AIMS was able to create a framework necessary for training AI algorithms. They functioned as critical precursors to AI by making the large amounts of organized data necessary for predictive modelling and decision aid systems [2–4,8]. The evolution from rule-based systems to contemporary deep learning networks reflects a paradigm shift in how anesthesiologists approach perioperative care [34]. For example, machine learning algorithms highlight how early decision-support systems laid the foundation for today’s predictive AI models.

More recently, AI evolution has been marked by several milestones, including the application of forecasting intraoperative and postoperative events, real-time decision-making help, and ultrasound image analysis in regional anesthesia. These developments showcase a gravitation from retrospective data analysis to prospective, assistive technologies that improve clinical efficacy and risk reduction [35]. Similarly, using AI to help in critical care decision making, such as ventilator weaning, shows the true extent and power of the role of AI in bedside care [35].

Overall, AI’s journey in medicine, more specifically anesthesiology, reflects a broader transformation from data/record documentation to data-informed clinical care. As the world progresses and more technologies are created, the absolute essential role of AIMS has shown us that digital infrastructure has enabled AI to become an absolute essential component of contemporary perioperative medicine. New deep learning tools, AI tools that are trained under a large amount of data, utilize self-attention layers, aiding AI to focus on key information and achieve better performance than older models like recurrent neural networks (tracking changes over time) and convolutional neural networks (reading images) [36].

## Application of AI in Anesthesia

### Patient Monitoring and Intraoperative Decision Support

AI applications in anesthesiology now work across real-time patient monitoring, pharmacologic optimization, and closed-loop systems, underscoring their cross-phase utility throughout the perioperative workflow [37]. AI models supporting intraoperative decision-making are especially useful in continuous physiologic analysis, modifying therapeutic approaches, and increasing intraoperative safety margins [38]. One of its main applications is the constant monitoring and analysis of vital signs. Commonly used monitoring systems display physiological data, but what AI platforms do is recognize patterns and subtle changes that are critical to the patient’s health and can influence providers to make quick, life-saving decisions. For example, machine learning algorithms have been developed to predict intraoperative hypoxia, hypotension, and other adverse events before they are evident on physical examination, allowing for earlier corrective measures [38,39]. Similarly, AI systems are also being used in intensive care unit settings, where real-time monitoring aids in preliminary interventions in cases like septic shock and respiratory failure [38]. The role of AI in predicting intraoperative hypotension using reliable algorithms, emphasizing how these systems can provide anticipatory signals that enhance an anesthesiologist’s response and patient safety [39]. Other researchers have reported that neural-network–based systems like convoluted models have now outperformed our typical tools in perioperative risk prediction, resulting in area under the curve (AUC) scores greater than 0.92 for mortality foreshadowing [40]. Acute kidney damage (AKI), a frequent postoperative problem that can dramatically raise morbidity, and even intraoperative awareness using EEG signal analysis were among the issues that these models demonstrated promise in predicting [40,41]. It was found that using a model that distinguishes short-term changes from long-term ones regarding a patient’s vital signs made it more convenient to forecast serious outcomes like ICU death, reaching an AUC (area under the curve) of about 0.92 [42]. It has been shown that computerized learning can precisely predict health conflicts like AKI, heart attacks, and reintubation utilizing pre- and intraoperative data. To further evaluate AI’s role in a variety of clinical settings, AI models were utilized across multiple hospitals, achieving AUCs of 0.79–0.86 [43]. To prevent complications like hypotension, postoperative nausea and vomiting (PONV), and even anesthesia awareness, Zhang et al. [40] describe how supervised learning models, such as support vector machines (SVMs), which categorize complex data by determining the optimal boundary between outcomes, and neural networks, have been trained on intraoperative data such as vital signs, demographics, and drug dosing [39,40]. However, the implementation of AI in healthcare is not without significant hurdles and challenges [44].

Another major advancement is the use of AI in closed-loop anesthetic delivery systems. Closed-loop anesthesia delivery systems are tools that automatically adjust the amount and delivery of anesthetic drugs based on the real-time physiological data from the patient, such as their blood pressure, heart rate, oxygen saturation, or depth of anesthesia, maintaining optimal levels of sedation and analgesia while minimizing drug-related complications. Closed-loop systems are an autopilot for anesthesiologists; the systems monitor the patient and make continuous changes to keep the patient properly sedated [13]. Closed-loop systems such as BIS-guided propofol infusion platforms showcase how AI can precisely measure anesthetic depth based on consistent real-time feedback [13]. In anesthesiology, Bitkina et al. [45] highlight that closed-loop and AI-driven machinery not only minimize diversity and maintain physiological goals but also cover applications in anesthesia, intravenous fluids, vasopressors, ventilation, and glucose control, depicting a wide range of clinical applications and better results [45]. In their pursuit of full-cycle automation, researchers have emphasized how closed-loop platforms are expanding beyond sedation to include predictive analytics for occurrences such as intraoperative hypotension [46]. The Bispectral Index is an EEG monitoring tool that aids an anesthesiologist in telling what level of sedation the patient is in [47]. The growing reliability of Bispectral Index (BIS) guided systems and target-controlled infusions is notable, among the initial usages of AI successfully supporting precision anesthetic titration in live patient care [23]. To further elaborate on this, describing how AI-driven closed-loop systems—mostly those using EEG and BIS metrics—can create anesthetic dosing in real-time while mitigating human error [48].

Tools like this show the potential to enhance not only patient outcomes and clinical efficacy but also reduce the mental burden on anesthesiologists during difficult surgeries. Overall, these AI applications reflect a shift from defense to offense in proactive intraoperative care, where predictive algorithms and automation support anesthesiologists in maintaining patient stability and improving outcomes throughout the perioperative period [36]. Physician hesitancy and skepticism remain huge barriers to AI adoption, necessitating targeted education and simplified model explainability are necessary for integration into healthcare [44,49].

### Risk Prediction and Preoperative Assessment

Artificial intelligence has shown great promise in enhancing preoperative assessment and risk stratification. By using large datasets and machine learning techniques, helpful predictive models have been made to assess the probability of complications before surgery. Scientists have also discussed how AI tools are being integrated into early perioperative workflow, such as pre-operative assessments, thus aiding in American Society of Anesthesiolgoists (ASA) classification and stratifying ventilator strategies for high-risk patients [50]. ASA classification is a scale used by anesthesiologists to gauge how risky anesthesia may be for a patient, it is a 1 to 6 scale [50].

For example, AI algorithms are being used to foresee difficult airways, help with ASA classification, and ICU admission or extended hospital stay [51].

These aides can analyze a broad spectrum of variables, for instance, like demographics, comorbidities, prior surgical outcomes, and lab values, helping us identify patients at higher risk with greater accuracy than our traditional past scoring systems. This lets an anesthesiologist make highly informed decisions during surgical planning and aids in allocating resources [52]. Researchers describe a predictive system that incorporates physiologic patient data with natural language processing (NLP) from clinical charting to improve preoperative risk stratification. This technique would allow for a mitigation of predictive risks, allowing anesthesiologists more time to cultivate specific care plans for complex patients [52].

It was also observed that Gemini, a publicly available AI tool, aligned with anesthesiologists’ spinal anesthesia decisions in 68.5% of 72 cases, with even greater agreement—85.7%—among patients on medication. These findings suggest its value as a supportive aid in preoperative planning, enhancing decision-making while preserving clinician oversight [53].

More specifically, in this context, artificial intelligence acts as an enhancer of the clinical support system, giving us real-time data that can aid physician judgment as well as improve overall perioperative care. AI has also shown promise in evaluating fluid responsiveness. Researchers have found that AI-assisted ultrasound accurately assessed Inferior Vena Cava collapsibility and decreased variability between subcostal and transhepatic views, aiding in its use for perioperative hemodynamic monitoring [54]. Overall, incorporating artificial intelligence into preoperative care not only supports individualized risk prediction but also helps with time management, allowing for a more organized workplace and time for personalized anaesthesia planning [13].

### Personalized Anesthetic Management

Artificial intelligence is becoming increasingly applicable to customizing anesthetic care, particularly through patient-specific drug dosing and decision-making driven by neural networks. These systems integrate complex patient data—such as age, weight, comorbidities, and physiological responses—to enable more precise and adaptive anesthetic management in real time [55].

Predictive models and machine learning have shown promise in tailoring anesthetic plans informed by comorbidities and genetic factors. AI systems have been used to support pharmacogenomics-informed anesthesia, helping clinicians anticipate how patients will respond to various anesthetic agents and dosages [56]. Researchers have showcased that utilizing patient genetic information, such as CYP450 polymorphisms, in anesthetic planning can enhance drug selection and dosing, exploring the concept of pharmacogenomics-guided anesthesia [56]. This precision-based approach with a new “human-in-the-loop” model that uses both AI-based analgesia predictions with real-time clinician input, optimizing accuracy and custom care in pain management during surgery [57]. AI tools are being spearheaded in perioperative pain management; although these systems support a shift toward tailored care, their broader usage is still quite limited by verification discrepancies and provider hesitancy [58].

Initiatives to bridge this gap include the Safe Brain Initiative (SBI). To improve transparency and patient-centered quality assurance, SBI encourages cooperation among anesthesiologists, surgical teams, and quality monitors to integrate EEG-based monitoring, standardized reporting metrics, and patient-reported outcomes (PROs) into perioperative protocols [59].

In one study, learning algorithms analyzing EEG data were able to classify sedation depth more accurately than traditional methods, demonstrating their utility in intraoperative anesthetic titration [60]. AI models using functional MRI data could track brain network activity to estimate anesthetic depth. This approach offers a non-invasive way to observe consciousness levels more precisely than EEG alone, showcasing AI’s role in both anesthesia management and understanding brain function during analgesic application [60]. AI-aided monitoring tools using EEG and hemodynamic limitations are advancing toward more custom anesthetic titration in both general and regional anesthesia.^12^ Regarding regional anesthesia, AI-guided ultrasound machinery is being created to improve nerve localization, needle placement, and block success rates, creating the way for a more standardized procedure technique [61].

Additionally, AI-driven dosing systems have been implemented in procedures such as gastrointestinal endoscopy, where deep neural networks successfully estimated anesthetic needs using real-time physiologic and biometric data with high recall and accuracy [62]. These systems represent a step toward real-time, closed-loop anesthesia delivery, supporting safer dosing and reducing the risk of both under- and over-medication [62]. More recent studies have depicted how artificial intelligence plays a part in pain profiling, where machine learning models use clinical, radiological, and psychological data to distinguish between sorts of chronic pain and guide customized pain management plans [63].

While these approaches are still undergoing validation, current evidence suggests that AI can enhance anesthetic precision by accounting for individual variability, ultimately advancing the field toward more personalized and predictive perioperative management [64].

### Operating Room Efficiency and Workflow Optimization

**Figure 1:**
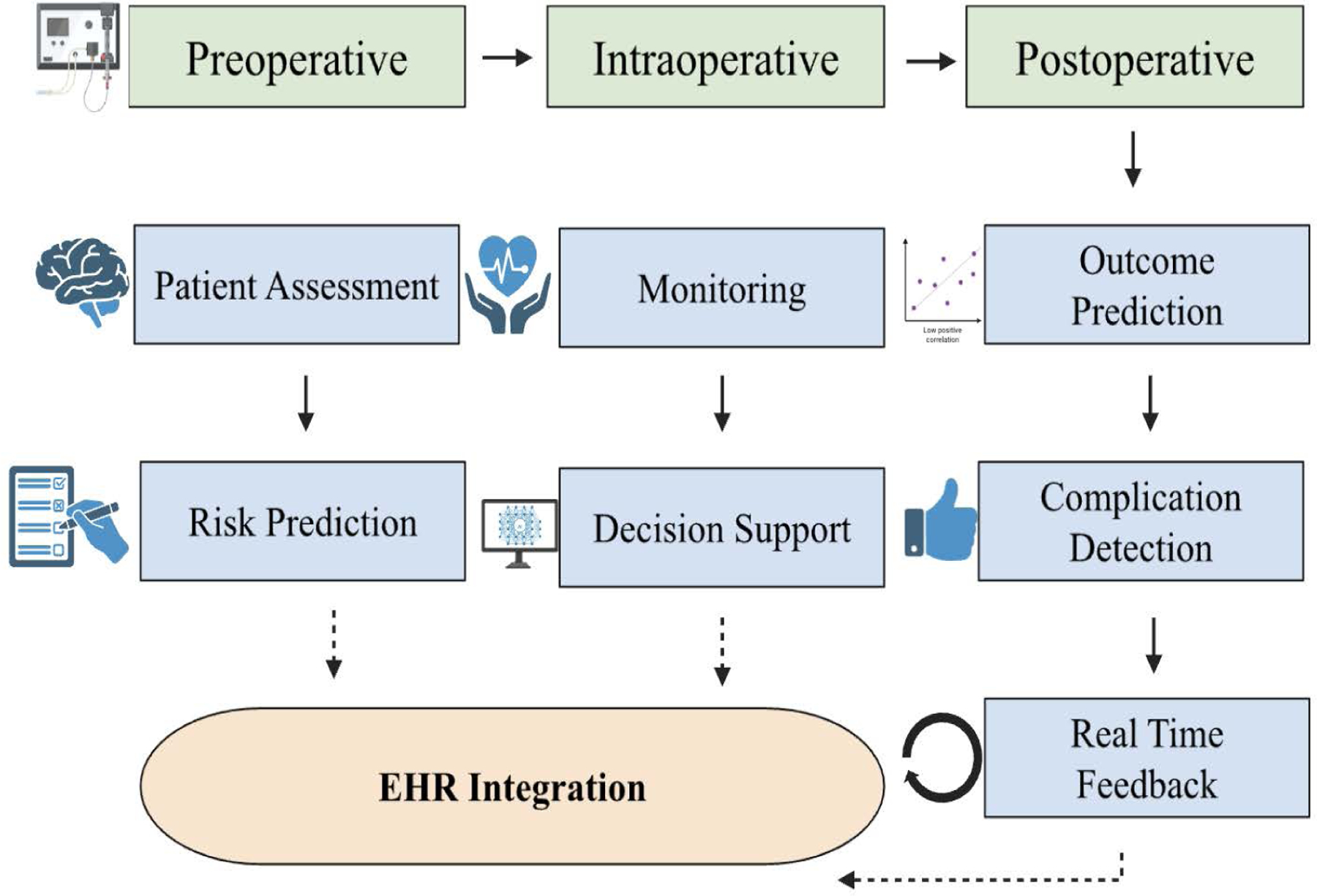
An overview on the workflow in the Artificial intelligence (AI) in Anesthesia: This figure depicts the incorporation of artificial intelligence across the surgical realms, the preoperative, intraoperative, and postoperative phases. AI aides such as patient assessment, real-time monitoring, and outcome prediction support difficult decision-making through risk qualification, clinical support, and risk detection. These domains are used in combination with the electronic health record systems (EHRs), letting people continue to learn and create closed-loop feedback systems. This framework, spearheaded by AI, allows tailored and data-backed care, as well as precise anesthetic administration.

Artificial intelligence is being used to better operating room (OR) efficiency by streamlining scheduling, coordinating staff in a more organized manner, and reducing the turnover time between patient cases. AI-supported decision systems can recognize patterns and trends, patient-specific risks, and even schedule staff to manage resource allocation [55]. These aides can help minimize delays, increase OR usage, and improve overall perioperative flow. The workflow optimization remains a primary advantage of AI systems, more specifically when applied to OR organization, communication streamlining, and documentation computerization, all of which improve team communication and minimize administrative overhead [18]. AI’s role in OR logistics is emphasized, including machine case prioritization, adaptive scheduling, and intelligent alert systems. These tools collectively aim to organize case turnover and reduce administrative workload [34] (Figure 1).

In addition, AI-centered platforms that incorporate natural language processing (NLP) are being developed to automate parts of intraoperative and postoperative record keeping. These systems can extrapolate key clinical data from operative notes or verbal dictation and transform them into structured records, thus minimizing the cognitive burden and clerical time for physicians [24]. While still in its premature stages of clinical integration, NLP and speech recognition tools have shown great promise in their pilot settings to create anesthesia records and promote more complete documentation.

Overall, by introducing a predictive model for scheduling with documentation tools, AI has the potential to majorly improve the OR protocol and enhance communication among perioperative teams, allowing anesthesiologists to devote more of their cognitive ability to make high-focus clinical decisions. These organizational domains aid junior staff in making decisions that are like more experienced providers.

## Ethical, Legal, Educational Considerations

As artificial intelligence systems have become increasingly incorporated into anesthesiology, legal, ethical, and educational challenges have arisen that must be addressed alongside technological progression. A main concern is the lack of an algorithm disclosure —often called the “black box” problem, limiting a physician’s ability to analyze or trust AI-generated recommendations, notably in high-risk environments, much like the operating room [24]. Clinical reasoning under high-stress environments is susceptible to cognitive overload, causing provider stress and constant shifting from quick, intuitive thinking to more error-prone methods; AI tools can step in and can recognize major trends and filter non-relevant information [65]. This vagueness also raises accountability questions. In cases where AI partakes in a medical error, the legal obligation—whether borne by the clinician, software developer, or institution—remains unclear and is poorly defined [55]. Ethical usage of AI necessitates transparent trial design, including the clear underscoring of responsibility, fair patient choice, and informed consent structures that mostly address the intricacies of AI-driven clinical care [36]. Ethical AI usage in anesthesiology must include informed consent, algorithm explanation, and the protection of clinician autonomy in decision-making [66]. About 70% of anesthesiologists in one study voiced concern about legal responsibility if AI-led decisions caused harm [17]. Defending physician autonomy is vital to keep away from blind reliance on AI technologies and ensure that medical decisions remain supported by human clinical judgment and patient data [15]. Another author has raised concern about automation bias, where doctors may blindly follow AI recommendations, and warn about algorithmic drift; whereas newer models may transform over time, potentially compromising safety if not critically verified [67]. A complementary methodological perspective comes from researchers who analyzed the use of AI tools, especially ASReview (Active Learning for systematic reviews), for systematic reviews. They discovered that while AI can reduce workload—screening only 23 % of records—it also brings in risks like bias due to single-reviewer training, duplication conflicts, and subpar reporting of screening verdicts [68].

Patient data privacy is another significant issue in this regard. On top of privacy, data diversity and algorithmic bias in critical care environments create a risk to prediction validity and physician trust, especially when AI domains are primed on non-standardized inputs [20]. AI systems depend heavily on massive datasets typically gathered from electronic health records, which causes concern for data security, informed consent, and adherence to regulations like HIPAA compliance with regulations [28,35,69]. Physicians have expressed concern about how data might be repurposed without patient awareness, more specifically in cross-border digital platforms.

**Figure 2:**
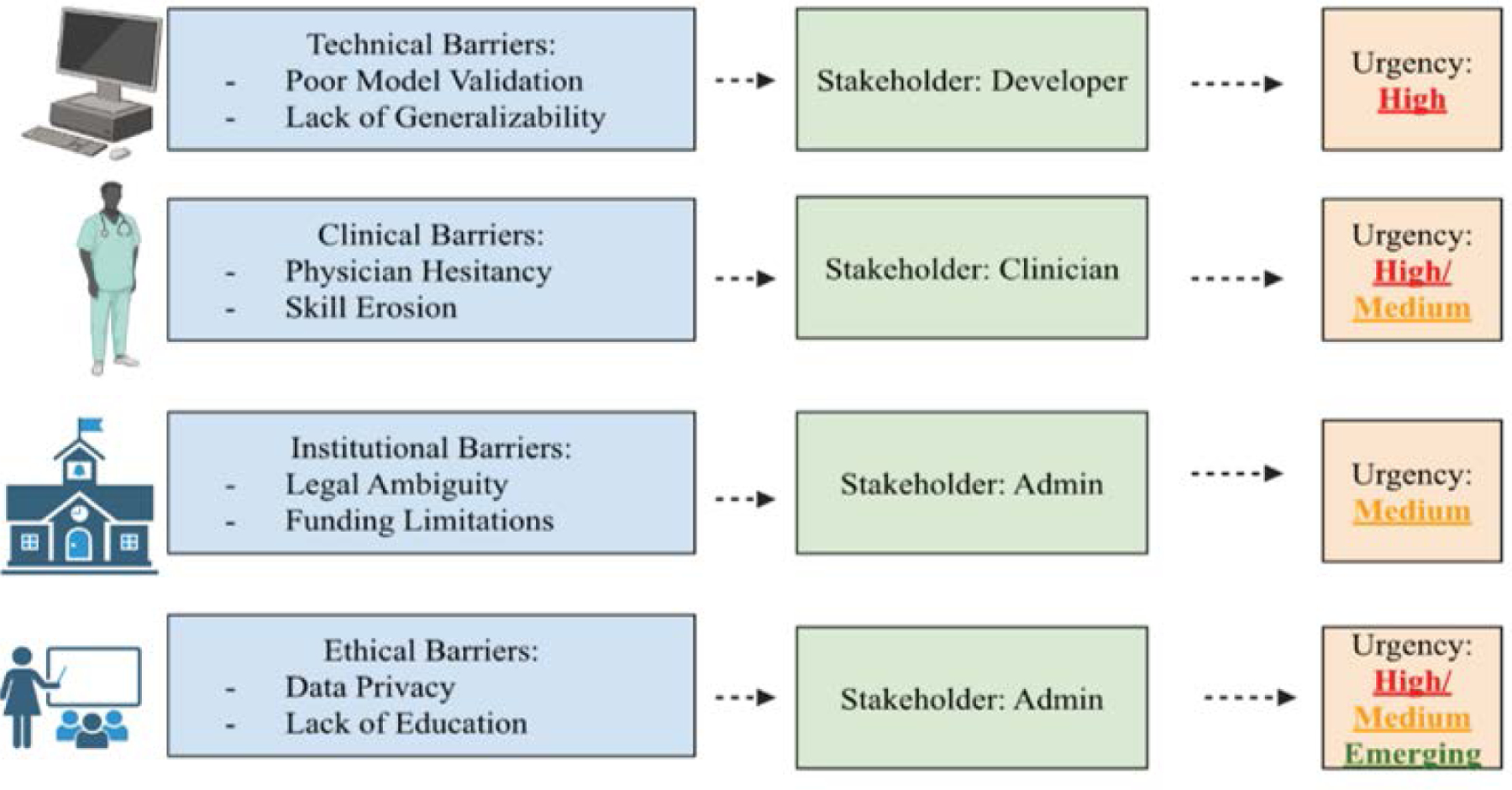
This diagram depicts the main stakeholder group, domain, and urgency of the obstacles to the integration of artificial intelligence (AI) in anesthesiology. The four main categories in this table—technical, clinical, institutional, and ethical/societal—are each linked to examples. Stakeholders highlight the main parties involved in tackling each concern, while urgency levels (high, medium, and emerging) represent how serious each one is. The multifaceted character of AI adoption in perioperative care is highlighted by the contrast of practical challenges like limited model generalizability and unknown responsibility with ethical considerations like algorithmic bias and data privacy.

From an educational standpoint, the swift evolution of AI tools requires the incorporation of AI literacy into medical training. AI-generated color covers during ultrasound-guided regional anesthesia significantly enhanced junior acquisition of proper sonoanatomical views and their ability to retain these skills even two months after teaching, according to a recent randomized study; this suggests AI could strengthen procedural learning long after initial instruction [70]. It has been noted that anesthesia training programs must adapt to include both technical use of AI tools and critical thinking about their integrations, cons, and ethical concerns [23,35]. Studies have shown that although many anesthesiologists are aware of AI, a large portion are not familiar, depicting a surge in academic and clinical interest in the field [37]. This knowledge gap is similarly discussed by other researchers, who call for stronger AI-focused medical education and emphasize that without clinicians’ understanding, adoption rates may plateau regardless of technological progression [71] (Figure 2).

There is an evident hesitation to incorporate these tools into clinicians’ practice, especially due to unfamiliarity [41]. Clinician hesitation and skepticism as large barriers to AI adoption and are reasons for targeted education and improved model explainability in the physician domain [36]. Advocacy for a multi-pillar strategy that uses clinician education, regulatory clarity, and transparent model design for the safe integration of AI in anesthesiology [31,72,73].

Clinicians’ trust in AI systems heavily relies on explainability and accountability, specifically in high-stakes environments like the operating room [32]. Amplifying these concerns, Webster et al. [74] highlight the challenge of clinician trust in AI systems, stressing the importance of explainable models for safe clinical use [74]. Additional concerns about overreliance on AI include the fact that it may lead to a decrease in clinician expertise and loss of situational awareness during critical periods, especially if human oversight is not maintained [39]. AI tools must be analyzed not only on their activity but also within the technical systems they operate in, as ignoring human–technology interaction may synthesize modern forms of risk in perioperative care [75].

## Limitations and Barriers to Clinical Implementation

Despite its revolutionary potential, the clinical integration of AI in anesthesiology and perioperative medicine faces multiple limitations that stop its universal utilization. There are similar implementation barriers, including fragmented data ecosystems, hazy ethical standards, and a difference in clinician readiness across different practice environments [30]. The vital importance of analyzing failure and patterns in anesthetic medication paves the path to routine success. Härkänen et al. [76] state that their Safety-I and Safety-II framework shows how everyday clinical workflows, such as standardized preparation, adaptive teamwork, and cross-checking, all contribute to patient safety. This tells us how AI must be used as a supportive measure, not a standalone aid [76]. Even though there are promising outcomes in controlled studies, AI aides often face a substantial integration gap in real-world perioperative environments, showcasing, once again, the need for regulatory alignment and clinician involvement in model development [38]. The anesthesiology field of medicine faces many workforce risks, such as substance abuse, chronic stress, general exposure to radiation, and anaesthetic gases that can hinder providers’ ability to integrate AI tools within their fields in terms of reducing awareness about their surroundings, thus increasing error [77]. We must also recognize the educational efforts needed to improve AI literacy in remote or underserved environments; connecting this digital divide is essential to guarantee equitable AI integration in universal healthcare [78]. Even though, there has been a surplus of tech innovation, the general field of anesthesiology, like any other field still has conflicts with typical workplace issues such as team-based communication and unsatisfactory safety lineup; to see proper improvement within the field, both general field improvements must be made in parallel with AI to proportionately increase patient safety [79].

Contemporary AI applications often succumb to clinical expectations due to a lack of prospective trials or studies, segmented validation efforts, and discrepancies between developers and frontline users [31]. This issue has been underscored by cautioning that many studies focus on AI’s diagnostic accuracy without hard evidence of direct patient benefitting, and stress that future randomized controlled trials are necessary for validating AI tools in real-world clinical environments [80]. Another point of contention includes intraoperative MRI (iMRI) hybrid operating rooms, issues like signal interference, difficulty reaching the patient, and trouble with communication. These specific setups are not typically considered when AI tools are being made, emphasising the importance of testing these AI aides in real-world surgical environments [81]. Scientists emphasize that although AI tools have demonstrated potential, their clinical integration remains hindered by a lack of real-time validation studies and minimal adoption into actual anesthetic environments [82]. This sentiment is also supported by other researchers who found that a surplus of AI clinical trials does not meet ethical research standards, including scientific validity and risk–benefit comparison, potentially undermining both efficacy and adoption [43].

A key barrier to AI integration in anesthesiology is the lack of standardized validation frameworks across institutions, which limits the external applicability of many contemporary models [19]. The same sentiments are emphasized by the criticalness of data standardization universally to ensure model generalizability and reproducibility, warning that differing EHR systems and a lack of shared data domains hinder AI scalability [18]. The importance of rigorous validation and patient-cantered implementation strategies is emphasized, warning that unverified models may cause unexpected safety risks in high-pressure perioperative settings [83]. Scientists have discussed how perioperative AI systems are trained on past data without external validation, making them at risk of performance decreases in real-world scenarios due to population and methodology [26]. It has been shown that computerized learning can precisely predict health conflicts like AKI, heart attacks, and reintubation utilizing pre- and intraoperative data. AI models were utilized across multiple hospitals, achieving AUCs of 0.79–0.86, which highlights AI’s role in a variety of clinical settings [41].

It has been shown that computerized learning can precisely predict health conflicts like AKI, heart attacks, and reintubation utilizing pre- and intraoperative data. A primary concern is its algorithm’s generalizability across distinct patient populations and healthcare models across the world. Many AI models are trained using data from certain institutions or countries, causing concerns about their credibility and efficacy when applied to patient care settings with a variety of demographics and procedures [55].

In addition, another obstacle is the dependence on organized, high-quality electronic health record (EHR) data. AI systems often rely on transparent, regulated, and whole datasets to work properly. In the real world, however, medical record keeping may contain flaws and be unorganized, hindering the algorithms’ performance and escalating the risk of faulty outputs. In line with this, the lack of cohesive data standards and the variability in EHR structures across institutions may compromise algorithm validity and reproducibility [34]. They advocate for greater investment in data synchronization frameworks to overcome these hurdles. Inconsistent usage of tools like barcode scanning, electronic prescribing, and medication double-checks raises the risk of errors, but suggests that AI could help close this gap through real-time alerts, natural language processing, and computerized error detection [84]. AI model deployment is often hindered by a lack of generalizability and the need for contextual clinician training, both of which are critical for adoption in anesthetic practice [85]. Clinical apprehension remains a significant obstacle. This sentiment is emphasized, explaining that although AI models can synthesize knowledge or aid in documentation, they are not yet verified for intensive intraoperative care due to concerns over hallucinated content, patient well-being, and lack of physician approval [86]. A surplus of anesthesiologists displays caution regarding AI tool integration, claiming a lack of optimal clinical trials and a limited explanation of algorithmic decision-making [72]. This hesitation is exaggerated by concerns about medical liability, especially in the possibility that there could be adverse outcomes driven by AI. Finally, administrative and funding obstacles hamper the scalability of AI innovations. Legal frameworks are still in process, and approval pathways for AI in the field of anesthesiology remain clouded in mystery. There is also the issue of limited institutional funding and a lack of reimbursement, hindering invention in AI development and integration. Together, these obstacles showcase the need for a clear model design, refined data infrastructure, and greater cross-disciplinary collaboration to confirm safe and equitable integration of AI into clinical routine practice.

## Future Directions and Research Opportunities

As artificial intelligence becomes increasingly incorporated into anesthesiology, future directions must enhance analysability, broader clinical incorporation, education, and verification through high-level evidence. Even though there have been recent advances, several areas of artificial intelligence necessitate more exploration and progression. Researchers analyzed 161 systematic reviews on AI in clinical medicine and discovered that just a small percentage of them described model training procedures or performance measures, and less than half analyzed the potential of bias using AI-specific tools [87]. The clinical usefulness and reproducibility of AI across specialties are weakened by this absence of consistent reporting [87]. Researchers evaluated over 160 AI-focused systematic reviews and realized widespread discrepancies in announcing model design and verification procedures [88]. Their proposed CLASMOD-AI framework showcases a step toward creating a more transparent analysis and reporting standard for AI in clinical care [89].

One of the primary concerns regarding AI integration is the need for interpretable” or “white-box” AI models, allowing physicians with clear reasoning behind AI’s diagnostic predictions. Currently, most AI algorithms function as “black boxes,” which instill confidence and incorporation due to the lack of explanation [90]. Understandable AI frameworks attempt to close this gap by allowing anesthesiologists to truly grasp how certain variables contribute to the decision-making processes, thereby improving accountability and clinical confidence [55].

In addition, another major opportunity remains in enlarging AI applications in regional anesthesia, telemedicine, and critical care. Decision support tools may refine ICU triage, sedation convention, and ventilator titration, provided data quality and workflow streamlining conflicts are addressed [20]. Scientists have proposed expanding AI use into perioperative pain management specifically, suggesting that predictive models could help anesthesiologists better titrate analgesics, monitor pain trajectories, and minimize opioid-related complications [23]. More specifically, for regional anesthesia, AI-assisted ultrasound interpretation has depicted promise in enhancing anatomical landmark identification and block accuracy [23]. Robotic intubation domains and AI-assisted nerve block insertions have been discussed to enhance procedural efficiency and accuracy [28]. The merging of AI into telemedicine platforms may also improve perioperative consultation, virtual monitoring, and access to care in underserved regions.

In healthcare education and simulation, AI-driven tools are being made to progress ultrasound-guided procedures, manage airways, and sedation planning. AI-enhanced simulators are hoped to be used for regional anesthesia training, where real-time feedback and anatomical recognition algorithms help in making novice anesthesiologists more comfortable with their procedural confidence [61]. AI-enhanced simulators are hoped to be used for regional anesthesia training, where real-time feedback and anatomical recognition algorithms help in making novice anesthesiologists more comfortable with their procedural confidence [18]. Advocacy for early incorporation of AI literacy into clinical training, along with simulation-based learning to ensure safe, confident adoption by future anesthesiologists [74]. Although AI-based tools hold immense promise for customs anesthetic delivery, their efficacy remains contingent on intense validation and clinician training to guarantee safe incorporation [91,92]. Future integration may lean on models that primarily support mixed decision systems that enhance AI through direct anesthesiologist feedback, connecting human expertise with machine precision [57].

Machine learning-enhanced simulators can adjust to trainee performance, thus giving them tailored feedback, an extensible approach to skill development. In addition, in the field of anesthesiology, researchers have been combining virtual reality and AI to help create a learning environment for very specific rate-critical events like anesthetic system toxicity.

To ensure the safety and efficacy of these AI tools, the field must prioritize large-scale, multicenter future trials that assess AI performance in a variety of clinical settings. Advocacy for the integration of ethical review criteria—such as justice, data control, and trial transparency—into AI development systems can aid in guaranteeing innovation and protecting patient contentment [43]. Most contemporary studies are retrospective and occur in single centers, thus hindering generalizability [4]. It could be difficult to translate the efficacy of AI therapies to actual clinical practice because most studies are single-center, lack patient-centered outcome measures, and have low demographic diversity, even though many of them indicate good diagnostic performance [4]. Advocacy for the integration of ethical review criteria—such as justice, data privacy, and trial transparency—into AI development systems can aid in guaranteeing innovation and protecting patient welfare [43].

In addition, trials must assess not only accuracy but also clinical impact, improving workflow, and promoting healthy patient outcomes. Without thorough corroboration, even the most advanced algorithms risk becoming misused.

In conclusion, the future of AI in anesthesiology heavily depends on the development of understandable systems, expansion into unexplored branches like regional anesthesia and critical care, use in simulation-based education, and reliable clinical evidence. With the prioritization of these elements, AI has the potential to become a life-changing force in perioperative medicine. Echoing this, advocacy for explainable AI frameworks and multi-center validation efforts that can bridge the gap between technological promise and bedside adoption is in the works [21].

AI systems manage intricate, dynamic environments, making them useful for high-pressure domains like intraoperative care, where rapid data analysis is vital [50]. Artificial intelligence is increasingly valuable in supporting real-time clinical decisions during surgeries.

## Conclusion

Artificial intelligence (AI) has shown great potential to transform anesthetic care by improving clinical decision-making, enhancing perioperative efficiency, and supporting personalized patient management [55]. Ranging from real-time monitoring and predictive metrics to AI-assisted ultrasound and workflow optimization, these technologies are already beginning to shape the future of anesthesiology.

As the field progresses, it is essential to prioritize responsible innovation. AI incorporation in anesthesiology requires not only technological innovation but also interdisciplinary trust, regulatory clarity, and transparent model development [74]. This includes promoting interdisciplinary collaboration, guaranteeing transparency in algorithm design, and recognizing the limitations of generalizability, data morality, and clinician certainty [92,93]. AI should complement—not replace—clinical wisdom. This concept is reinforced by those who argue that AI must serve as an assistant in the OR, mostly in critical or specific cases [48]. This view is echoed in the surgical field, highlighting AI’s role as a supportive agent, enhancing decision-making through shared intelligence, rather than physician substitution [94].

Maintaining a delicate balance between machine and human judgment is vital to protecting patient safety and advancing ethical integration in practice. Efforts must be made to prevent deskilling and strengthen human oversight in intraoperative care. As AI becomes more capable, physicians must actively protect their clinical intuition and conserve a humanistic presence, mostly in emotionally intelligent or ethically ambiguous patient cases. As medical students and future physicians, understanding both the possibilities and constraints of AI permits us to participate thoughtfully in its application and advancement for tools that authentically improve patient care. Future anesthesiologists must be versatile in interpreting algorithmic output as they are managing analgesics, showcasing a contemporary hybrid skill set vital to present-day practice.
